# Factors Associated With the Syndromic Diagnosis of Sexually Transmitted Infections Among Adolescent Girls and Young Women (AGYW): Findings From the Namibia DREAMS Program

**DOI:** 10.1155/jotm/8628807

**Published:** 2026-09-29

**Authors:** Enos Moyo, Endalkachew Melese, Hadrian Mangwana, Simon Takawira, Bernadette Harases, Rosalia Indongo, Perseverance Moyo, Ntombizodwa Makurira Nyoni, Tafadzwa Dzinamarira

**Affiliations:** ^1^ College of Health Sciences, School of Nursing & Public Health, University of Kwa-Zulu Natal, Durban, South Africa; ^2^ Project HOPE-The People-to-People Health Foundation, Inc., Windhoek, Namibia; ^3^ Project HOPE Namibia, Windhoek, Namibia; ^4^ Medical Centre Oshakati, Oshakati, Namibia; ^5^ School of Health Systems and Public Health, Faculty of Health Sciences, University of Pretoria, Pretoria, South Africa, up.ac.za

**Keywords:** adolescent girls and young women (AGYW), associated factors, Determined Resilient Empowered AIDS-free Mentored and Safe (DREAMS) project, prevalence, Project HOPE Namibia (PHN), Reducing HIV Vulnerability: Integrated Child and Youth Health (reach PHN) project, sexually transmitted infections (STIs)

## Abstract

**Background:**

Approximately one million curable sexually transmitted infections (STIs) are contracted daily by individuals aged 15–49 years worldwide. The study aimed to evaluate the prevalence of syndromic STIs and associated factors among AGYW participating in the Determined, Resilient, Empowered, AIDS‐free, Mentored, and Safe (DREAMS) program implemented by Project HOPE Namibia.

**Methods:**

Data for this retrospective study were obtained from the DREAMS program, covering 2018 to 2024. The population for the study consisted of sexually active AGYW aged 15–24 years who were screened for STIs. Data were analyzed using IBM Statistical Package for Social Sciences (SPSS) Version 29 by employing chi‐squared tests and binomial and multinomial logistic regression.

**Results:**

Of the 27,992 participants in this analysis, 3184 (11.4%) were syndromically diagnosed with an STI (95% CI: 11.0%–11.8%). Participants less likely to have an STI diagnosis included those aged 15–19 years (AOR = 0.74, 95% CI: 0.68–0.81), pregnant or breastfeeding (COR = 0.12, 95% CI: 0.02–0.87), and those using family planning (AOR = 0.25, 95% CI: 0.23–0.29). Higher likelihoods of syndromic STI diagnosis were seen in recurrent postexposure prophylaxis users (COR = 2.71, 95% CI: 1.21–6.07) and those engaging in sex under the influence of alcohol or drugs (AOR = 1.93, 95% CI: 1.2–2.95).

**Conclusion:**

We recommend that the program intensify education on safe sexual practices and the consistent use of condoms to prevent STIs among high‐risk AGYW. Additionally, we recommend that AGYW refrain from taking alcohol and drugs.

## 1. Introduction

Approximately one million curable sexually transmitted infections (STIs) are contracted daily by individuals aged 15–49 years worldwide [1]. In 2020, approximately 374 million new infections were reported among individuals aged 15–49 years, involving the four curable STIs: Chlamydia, gonorrhea, syphilis, and trichomoniasis. Approximately eight million adults aged 15 to 49 worldwide are infected with the herpes simplex virus [1]. In 2019, the global incidence of symptomatic STIs among sexually active adolescents and young adults (AYA) aged 10–24 years was approximately 7% [2]. Sub‐Saharan Africa (SSA) had the highest incidence, with 51.6% of the infections occurring in females. Between 1990 and 2019, adolescents aged 10 to 14 experienced the largest increase in STI incidence [2]. A study in SSA indicated a self‐reported STI prevalence of 6.9% among adolescent girls and young women (AGYW) [3].

AGYW are at an increased risk of STIs owing to various behavioral and biological factors [4]. Some of the behavioral factors include engaging in high‐risk activities, such as having multiple or concurrent partners [5], condomless sex [6], sex under the influence of alcohol and drugs [7, 8], and difficulties in negotiating condom use, particularly in transactional and age‐disparate sexual relationships [9]. Additionally, AGYW are less likely to access and utilize sexual health services due to concerns about stigma [10]. AGYW are biologically vulnerable to *Chlamydia trachomatis* and human papillomavirus (HPV) because of reduced cervical mucus production and increased cervical ectopy [11].

STIs can result in various health consequences for AGYW. STIs, whether ulcerative or nonulcerative, significantly increase HIV acquisition risk through biological mechanisms [12]. Furthermore, while most individuals remain asymptomatic, long‐term complications related to sexual and reproductive health (SRH) encompass pelvic inflammatory disease, subfertility, and ectopic pregnancies [13]. Additional complications encompass cervical cancer attributed to HPV [14] and hepatocellular carcinoma linked to hepatitis B [15]. Mother‐to‐child transmission of STIs may lead to stillbirth, neonatal mortality, low birth weight, premature birth, neonatal sepsis, neonatal conjunctivitis, and congenital deformities [16].

Understanding the burden of STIs among AGYW is crucial for achieving control of the HIV epidemic, as this population is disproportionately affected by the disease. One cross‐sectional study conducted in Namibia using urine samples from patients revealed that AGYW aged 15–19 years had an 18.2% incidence of chlamydia and gonorrhea infections [17]. However, STIs are reported by symptomatic syndromes in Namibia, and there is no fine age and sex disaggregation of the data. We utilized data from the Determined, Resilient, Empowered AIDS‐free, Mentored, and Safe (DREAMS) program, which includes both the DREAMS project and the DREAMS component of the Reducing HIV Vulnerability: Integrated Child and Youth Health (Reach) activity, implemented by the Project HOPE Namibia (PHN)‐led consortia, to assess the prevalence of STIs and associated factors among AGYW at enrollment. Understanding the burden of STIs among this population group may aid in developing prevention strategies, potentially leading to a decrease in new HIV infections.

## 2. Methods

### 2.1. Study Design

This study involved a retrospective analysis of programmatic data from AGYW aged 15–24 years who received clinical services through the DREAMS program, including both the DREAMS project and Reach PHN activity, implemented by the PHN‐led consortiums from June 4, 2018, to July 31, 2023, and from June 31, 2023, onwards, respectively. This study used a secondary analysis of program data from the DREAMS database, portions of which were previously described by Moyo et al. [18]. The study focused on AGYW who were assessed for combination HIV prevention interventions, including pre‐exposure prophylaxis (PrEP).

### 2.2. Program and Population

#### 2.2.1. DREAMS Approach to Adolescent and Youth‐Friendly Health Services (AYFHS) Provision

To increase the uptake of integrated, high‐impact HIV clinical services for AGYW and their sexual partners, the DREAMS program has been implementing a blended service delivery approach. This approach comprises three modalities that together address the key barriers to the availability and access of youth‐friendly SRH/HIV services. Modality 1 focused on strengthening the bidirectional community‐facility referral linkage system in all DREAMS program implementation districts. Key activities included reviewing the existing system to identify areas for improvement, refining referral slips and service directories, assigning referral focal persons, orienting staff on the system, and tracking referrals through paper‐based and digital systems. The approach also facilitated referrals by providing transportation and leveraging existing collaboration mechanisms to coordinate activities and review progress. Modality 2 aimed to strengthen AYFHS within high‐volume health facilities that serve as referral hubs for the DREAMS program. Efforts included training service providers to deliver AYFHS with an emphasis on empathy and a nonjudgmental approach, hiring nurses, social workers, and peer educators, mentoring and supervision, distributing social and behavioral communication materials, fostering meaningful engagement with adolescents and young people (AYP), and creating a supportive, youth‐friendly environment through facility renovations or the placement of prefabricated structures. The DREAMS program supported AYFHS in 35 health facilities. Modality 3 involved facility‐linked outreach teams, including nurses, peer educators, and social workers, who traveled from high‐volume health facilities to surrounding communities to deliver most SRH/HIV services. The team provided HIV testing services, immediate antiretroviral therapy (ART) initiation, PrEP services, short‐acting contraceptives, condoms, postviolence care, and syndromic STI management. The teams visited group‐based platforms regularly to ensure AGYW received tailored clinical services. They used existing community structures (e.g., schools and churches), tents, and program vehicles to provide private and confidential services. AGYW were informed in advance about service schedules and available options via flyers, posters, local radio, social media, and program staff. Supplies were sourced from hub health facilities through the existing supply chain management system, and services were provided in accordance with national guidelines and documented in the Ministry of Health and Social Services (MOHSS)–approved registers. The team is mentored and supervised by nurse mentors and a program‐employed doctor.

#### 2.2.2. STI Services

Screening and managing STIs were integral components of the DREAMS core package. The DREAMS program’s support for the prevention and control of STIs included revising and updating national guidelines and training materials, conducting staff training on the management of STIs, increasing literacy about STIs among AGYW and their sexual partners, implementing routine STI screening at all entry points, and providing diagnosis and treatment of STIs using the syndromic management approach. All participants in the DREAMS program were screened for STIs by asking whether they had STI‐related symptoms. No tests were conducted to confirm the diagnosis, as Namibia uses a syndromic approach to diagnose STIs.

### 2.3. Data Source

Anonymized data were obtained from the DREAMS program databases, including the DREAMS project and the Reach PHN activity. The source documents were the PrEP client records and clinical registers. The data gathered during the program included programmatic information, participants’ sociodemographic characteristics, HIV risk factors, and STI screening outcomes.

### 2.4. Outcome Variable

The outcome variable for this study was “STI screening.” The responses to this statement were “Not screened,” “Screened, needs treatment,” “Screened, already on treatment,” “Screened, no treatment needed,” and “Refused.” Participants who declined screening and those who were not screened were excluded from this study. “Screened, needs treatment” and “Screened, already on treatment” received a code of “2,” whereas “Screened, no treatment needed” was assigned a code of “1.”

### 2.5. Explanatory Variables

Sixteen independent variables, grouped into participants’ characteristics and HIV risk factors, were considered in this study. The variables were selected based on their relevance and significance to STI occurrence.

#### 2.5.1. Participants’ Characteristics

The participants’ characteristics included age, marital status, number of biological children, district where the service was received, year of enrollment, months in the programs, pregnancy or breastfeeding status, and participation in safe space HIV prevention sessions. Age, recorded in the dataset as a discrete numerical value, was recoded into age groups “15–19” and “20–24.” Marital status was recoded as “single” and “married,” and months in the programs as “0–6,” “7–12,” “13–24,” “25–36,” and “> 36.” Pregnancy or breastfeeding status was coded as “pregnant,” “breastfeeding,” and “neither.” The number of biological children was recoded as “0 children,” “1–2 children,” and “≥ three children,” while participation in safe space HIV prevention sessions was coded as “yes” and “no.”

#### 2.5.2. HIV Risk Factors

HIV risk factors documented included the knowledge of a partner’s HIV status, consistency of condom use, recurrent postexposure prophylaxis (PEP) use, indulgence in sex under the influence of alcohol or drugs, the experience of sexual violence, having multiple or concurrent sexual partners, use of a family planning method, and consideration of self‐risk of HIV. All the responses were coded as “yes” and “no.” Recurrent PEP use was defined as taking more than one course of PEP within a year in this study.

### 2.6. Data Quality Assurance

A comprehensive description of the program’s data quality assurance is presented in another publication [18]. In summary, we automated BioID generation and implemented skip rules and validation checks in our digital system. District and regional teams conducted programmatic spot checks, data quality assessments, and field monitoring to ensure that the provided data met minimum quality criteria.

### 2.7. Criteria for Inclusion in Data Analysis

Among the 30,864 participants aged 10–24 years who received clinical services through the program between 2018 and 2024, we excluded those outside the 15–24 age group, leaving 28,934 participants. We then excluded 935 participants who were not sexually active, leaving 27,999 participants. After excluding seven participants with missing data, we analyzed the remaining 27,992 participants.

### 2.8. Data Analysis

Data were exported from DHIS2 to IBM Statistical Package for Social Sciences (SPSS) Version 29 for analysis. Nominal and ordinal data were analyzed using descriptive statistics, including percentages and frequencies. Chi‐square tests assessed the relationships between syndromic STI diagnosis and participants’ characteristics. Before performing logistic regression, we checked for evidence of clustering in the data using Akaike’s information criterion (AIC) and Bayesian information criterion (BIC). The results revealed that there was no substantial evidence of clustering in the data. Therefore, statistically significant characteristics identified through chi‐square tests were analyzed using binomial logistic regression to assess their association with syndromic STI diagnosis. Characteristics demonstrating statistically significant associations with syndromic STI diagnosis (*p*‐value < 0.05) in binomial logistic regression were subsequently included in multinomial logistic regression to estimate adjusted odds ratios (AORs).

### 2.9. Ethical Considerations

A comprehensive description of the program’s ethical considerations is provided in another publication [18]. To summarize, various Namibian ministries authorized the program, ensured that collected data remained private and confidential, and required all minors to provide consent, with their parents or guardians providing informed consent. AGYW of legal age of 18 years completed a consent form. We obtained a waiver for the secondary data analysis since we used anonymous programmatic data.

## 3. Results

### 3.1. Characteristics and Risk Factors of Participants

Twenty‐seven thousand nine hundred ninety‐two (27,992) participants were included in this secondary analysis. Most of these participants were aged 20–24 (*n* = 16,063; 57.4%), had no children (*n* = 23,265; 83.1%), were less than six months in the programs (*n* = 18,250; 65.2%), were not victims of sexual violence (*n* = 27,933; 99.8%), did not participate in the safe space HIV prevention services (*n* = 23,845; 85.2%), and were neither pregnant nor breastfeeding (*n* = 27,080; 96.8%). Additionally, most participants were not recurrent PEP users (*n* = 27,961; 99.9%), were not using any family planning method at enrollment (*n* = 19,849; 70.9%), did not know their partners’ HIV status (*n* = 14,986; 53.5%), never or inconsistently used condoms (*n* = 18,249; 65.2%), did not have sex under the influence of alcohol or drugs (*n* = 27,829; 99.4%), did not have multiple or concurrent partners (*n* = 27,550; 98.4%), and did not consider themselves at risk of HIV (*n* = 27,986; 99.9%). More details are in Table 1.

**TABLE 1:** Frequency distribution of characteristics and risk factors of participants.

Characteristics	Frequency *n* (%)
Age group (years)	
15–19	11,929 (42.6)
20–24	16,063 (57.4)
Number of biological children	
0	23,265 (83.1)
1–2	4503 (16.1)
≥ 3	224 (0.8)
Marital status	
Single	8773 (31.3)
Married	57 (0.2)
Missing	19,162 (68.5)
Participated in safe space HIV prevention sessions	
Yes	4147 (14.8)
No	23,845 (85.2)
District	
Katima	5278 (18.9)
Omuthiya	3362 (12.0)
Onandjokwe	3827 (13.7)
Oshakati	1381 (4.9)
Tsumeb	2382 (8.5)
Windhoek	11,762 (42.0)
Year of enrollment	
2018	352 (1.3)
2019	2626 (9.4)
2020	7795 (27.8)
2021	6758 (24.1)
2022	4461 (15.9)
2023	1984 (7.2)
2024	4016 (14.3)
Months in the program	
0–6 months	18,250 (65.2)
7–12 months	2016 (7.2)
13–24 months	2964 (10.6)
25–36 months	1697 (6.1)
> 36 months	3065 (10.9)
Using a family planning method	
Yes	8143 (29.1)
No	19,849 (70.9)
Partner’s HIV status is unknown	
Yes	14,986 (53.5)
No	13,006 (46.5)
Inconsistent or no condom use	
Yes	18,249 (65.2)
No	9743 (34.8)
A victim of sexual violence	
Yes	59 (0.2)
No	27,933 (99.8)
Recurrent HIV postexposure prophylaxis (PEP) use	
Yes	31 (0.1)
No	27,961 (99.9)
Having had sex under the influence of alcohol or drugs	
Yes	163 (0.6)
No	27,829 (99.4)
Has multiple or recurrent sexual partners	
Yes	442 (1.6)
No	27,550 (98.4)
Considers self at risk of HIV	
Yes	6 (0.0002)
No	27,986 (99.9)
Pregnancy or breastfeeding status	
Pregnant	848 (3.0)
Breastfeeding	64 (0.2)
Neither	27,080 (96.8)

### 3.2. Prevalence of Syndromic STIs Among Participants

Of the 27,992 participants included in this analysis, 3184 (11.4%) were diagnosed with a syndromic STI, with a 95% confidence interval (CI) (11.0%–11.8%). A subgroup analysis among 2686 AGYW who were PrEP users and returned for one‐month follow‐up visits revealed that 308 (11.5%) were diagnosed with a syndromic STI, with a 95% CI (10.3%–12.7%). There was no statistically significant difference in the prevalence of syndromic STIs at enrollment and after 1 month among those PrEP users.

### 3.3. Participants’ Characteristics Associated With STI Diagnosis

The chi‐square test results revealed that syndromic STI diagnosis was associated with age group, district, year of enrollment into the programs, duration in the programs, pregnancy or breastfeeding status, and safe space participation status (*p* < 0.05). Participants aged 15–19 years were less likely to be diagnosed with syndromic STIs than those aged 20–24 years, with an AOR of 0.74 (95% CI, 0.68–0.81). Participants in other districts were more likely to be diagnosed with a syndromic STI than those in Katima Mulilo. Participants who enrolled in 2018–2021 were more likely to be diagnosed with a syndromic STI than those who enrolled in 2024. In contrast, participants who enrolled in 2022 and 2023 were less likely to be diagnosed with a syndromic STI than those who enrolled in 2024 (AOR) = 0.34, 95% CI (0.25–0.45), and (AOR) = 0.32, 95% CI (0.22–0.48), respectively. Participants who were 13 months or more in the programs were less likely to be diagnosed with a syndromic STI than those with less than or equal to 6 months. However, participants who had been in the programs for 7–12 months were more likely to be diagnosed with a syndromic STI than those in the programs for 0–6 months, AOR = 2.38, 95% CI (2.12–2.69). Participants who had participated in the safe space HIV prevention sessions were more likely to be diagnosed with a syndromic STI than those who had not, AOR = 1.38, 95% CI (1.25–1.52). Participants who were pregnant or breastfeeding were less likely to be diagnosed with a syndromic STI than those who were neither pregnant nor breastfeeding (crude odds ratio [COR], 0.29; 95% CI, 0.20–0.41; COR, 0.12; 95% CI, 0.02–0.87, respectively). However, this relationship was not significant in the adjusted analysis. Additionally, the relationship between the number of biological children and syndromic STI diagnosis was also not significant in the adjusted analysis. More details are in Table 2.

**TABLE 2:** Participants’ characteristics associated with syndromic STI diagnosis.

Characteristics	Crude odds ratios	95% CI^∗^	Adjusted^∗∗^ odds ratios	95% CI^∗^	Chi‐square test *p*‐value
Age group (years)					< 0.01
15–19	0.73	0.68–0.79	0.74	0.68–0.81	
20–24	Reference	Reference	Reference	Reference	
Marital status					0.96
Single	NC	NC	NI	NI	
Married	NC	NC	NI	NI	
Number of biological children					< 0.01
0	Reference	Reference	Reference	Reference	
1–2	0.22	0.19–0.27	0.82	0.68–1.001	
≥ 3	0.18	0.08–0.41	0.74	0.32–1.68	
District					< 0.01
Katima Mulilo	Reference	Reference	Reference	Reference	
Omuthiya	2.52	2.19–2.90	2.58	2.21–3.02	
Onandjokwe	2.37	2.07–2.72	2.17	1.86–2.53	
Oshakati	0.92	0.73–1.17	2.64	1.97–3.55	
Tsumeb	2.56	2.20–2.99	1.81	1.53–2.14	
Windhoek	1.57	1.39–1.78	1.94	1.70–2.22	
Year of enrollment					< 0.01
2018	2.26	1.57–3.26	5.75	3.76–8.79	
2019	5.31	4.49–6.28	11.90	9.41–15.05	
2020	5.52	4.75–6.42	6.24	5.05–7.72	
2021	1.39	1.18–165	1.36	1.09–1.70	
2022	0.35	0.27–0.45	0.34	0.25–0.45	
2023	0.32	0.22–0.46	0.32	0.22–0.48	
2024	Reference	Reference	Reference	Reference	
Months in the programs					< 0.01
0–6 months	Reference	Reference	Reference	Reference	
7–12 months	4.20	3.79–4.66	2.38	2.12–2.69	
13–24 months	1.42	1.26–1.59	0.81	0.72–0.92	
25–36 months	0.19	0.14–0.26	0.07	0.05–0.10	
> 36 months	0.38	0.31–0.45	0.12	0.10–0.15	
Pregnancy or breastfeeding status					< 0.01
Pregnant	0.29	0.20–0.41	0.72	0.49–2.57	
Breastfeeding	0.12	0.02–0.87	0.35	0.05–2.57	
Neither	Reference	Reference	Reference	Reference	
Participated in safe space HIV prevention sessions					< 0.01
Yes	2.69	2.47–2.93	1.38	1.25–1.52	
No	Reference	Reference	Reference	Reference	

Abbreviations: NC, not computed; NI, not included.

^∗^CI is the 95% confidence interval.

^∗∗^Adjusted for age group, district, year of enrollment, number of months in the program, participation in safe space HIV prevention sessions, breastfeeding or pregnancy status, and the number of biological children.

### 3.4. Risk Factors Associated With Syndromic STI Diagnosis Among Participants

The results of chi‐square tests revealed that syndromic STI diagnosis was associated with the use of family planning, having sex under the influence of alcohol or drugs, recurrent PrEP use, condom use, being a victim of sexual violence, and consideration of HIV self‐risk (*p* < 0.05). Participants who used a family planning method were less likely to be diagnosed with a syndromic STI (AOR = 0.25; 95% CI, 0.23–0.29). Participants who never or inconsistently used condoms and those who considered themselves at risk of HIV were less likely to be diagnosed with a syndromic STI, AOR = 0.47, 95% CI (0.43 – 0.51), AOR = 0.81, 95% CI (0.75–0.88), respectively. In contrast, participants who had sex under the influence of alcohol or drugs and victims of sexual violence had a higher likelihood of being diagnosed with a syndromic STI, AOR = 1.93, 95% CI (1.27–2.95), AOR = 3.96, 95% CI (2.23–7.02), respectively. More details are in Table 3.

**TABLE 3:** Risk factors associated with syndromic STI diagnosis among participants.

Characteristics	Crude odds ratios	95% CI^∗^	Adjusted^∗∗^ odds ratios	95% CI^∗^	Chi‐square test *p*‐value
Using a family planning method					< 0.01
Yes	0.24	0.22–0.27	0.25	0.23–0.29	
No	Reference	Reference	Reference	Reference	
Partner’s HIV status is unknown					0.062
Yes	NC	NC	NI	NI	
No	NC	NC	NI	NI	
Inconsistent or no condom use	< 0.01				
Yes	0.42	0.39–0.45	0.47	0.43–0.51	
No	Reference	Reference	Reference	Reference	
Recurrent postexposure prophylaxis (PEP) use					0.01
Yes	2.71	1.21–6.07	2.29	0.98–5.33	
No	Reference	Reference	Reference	Reference	
Having had sex under the influence of alcohol or drugs					< 0.01
Yes	1.77	1.19–2.63	1.93	1.27–2.95	
No	Reference	Reference	Reference	Reference	
A victim of sexual violence					< 0.01
Yes	4.02	2.34–6.89	3.96	2.23–7.02	
No	Reference	Reference	Reference	Reference	
Has multiple or concurrent sexual partners	0.31				
Yes	NC	NC	NI	NI	
No	NC	NC	NI	NI	
Considers self at risk of HIV					< 0.01
Yes	0.61	0.56–0.66	0.81	0.75–0.88	
No	Reference	Reference	Reference	Reference	

Abbreviations: NC, not computed; NI, not included.

^∗^CI is the 95% confidence interval.

^∗∗^Adjusted for using a family planning method, condom use, recurrent PEP use, having sex under the influence of alcohol or drugs, being a victim of sexual violence, and consideration of HIV self‐risk.

## 4. Discussion

This study revealed that the prevalence of syndromic STIs among sexually active AGYW was 11.4%. Participants aged 15–19 years, with children, enrolled from 2022 to 2023, in programs for 13 months or longer, or who were pregnant or breastfeeding, exhibited a lower likelihood of syndromic STI diagnosis. In contrast, participants from all districts other than Katima Mulilo, who enrolled from 2018 to 2021, in the programs for 7–12 months, or who participated in the safe space HIV prevention sessions, were more likely to be diagnosed with a syndromic STI. Furthermore, participants who were on a family planning method, never or inconsistently used condoms, or considered themselves at risk of HIV were less likely to have a syndromic STI diagnosis. In contrast, recurrent PEP users, victims of sexual violence, or those who reported having sex under the influence of alcohol or drugs were more likely to be diagnosed with a syndromic STI.

The prevalence of syndromic STIs among AGYW was 11.4%, significantly higher than the 6.9% reported in a study conducted among the same population group in SSA [3]. The prevalence in this study may have been higher because AGYW who visited the DREAMS Program entry points are likely among the most vulnerable, placing them at a substantially higher risk of having a syndromic STI compared to the general population. In this study, there was no statistically significant difference in the prevalence of syndromic STIs at enrollment and after 1 month among those initiated on PrEP. This finding is at variance with earlier studies among PrEP users that showed an increase in STIs [19, 20]. This study’s findings may be attributable to health education on STIs and HIV offered to program participants. It should be noted that the syndromic approach is fairly accurate when identifying symptomatic STIs. However, a significant proportion of those infected usually do not have symptoms, so the approach cannot be used for them, resulting in the underdiagnosis of STIs. The use of cheap point‐of‐care tests, such as nucleic acid amplification tests (NAATs), which can detect *Neisseria gonorrhoeae*, *Trichomonas vaginalis*, and *Chlamydia trachomatis*, even among asymptomatic patients, should be encouraged in limited‐resource settings [21].

This study revealed that participants in Omuthiya, Onandjokwe, Tsumeb, Oshakati, and Windhoek had a higher likelihood of being diagnosed with a syndromic STI than those in Katima Mulilo. These findings align with an earlier Namibian study, which revealed that the Oshikoto and Khomas regions had higher STI prevalence than the Zambezi region [17]. These regional differences may be attributed to variations in AGYW’s culture, educational level, and socioeconomic status. Moreover, different economic activities in these areas, such as mining in Tsumeb, may contribute to STIs as people may reside in these areas without their regular partners or spouses.

This study revealed that participants aged 15–19 years were less likely to have a syndromic STI diagnosis than those aged 20–24 years. This finding is similar to that of a South African study [22]. This finding may be attributed to the tendency of young women aged 20–24 years to engage in multiple or concurrent sexual relationships, which increases their likelihood of contracting an STI [23]. In this study, AGYW with children exhibited a lower likelihood of having a syndromic STI diagnosis. This finding may be attributed to being in a stable relationship or being married, which reduced the likelihood of having multiple sexual partners, thereby reducing their risk of contracting STIs. Participants who enrolled from 2019 to 2021 exhibited a higher likelihood of syndromic STI diagnosis. Similar findings were reported in a South African study [24]. The findings may be attributed to the economic impact of COVID‐19, which likely increased the vulnerability of AGYW, resulting in heightened risks of sexual exploitation and child marriages [25].

This study revealed that program participants with 13 months or more of participation were less likely to be diagnosed with a syndromic STI. The observed finding may be associated with decreased risky behavior among participants, possibly attributed to the SRH education delivered during program activities. Reinforcing SRH information and STI prevention methods is essential to ensure the adoption of prevention measures. Participants in this study who were pregnant or breastfeeding were less likely to be diagnosed with a syndromic STI. The findings are plausible, as certain societies discourage sexual activity during the last trimester of pregnancy and the first 6 months postpartum [26].

Participants using any family planning method and those who considered themselves to be at risk of HIV in this study were less likely to be diagnosed with a syndromic STI. The use of condoms among participants may have influenced this relationship, as condoms are effective in preventing the transmission of STIs. The finding that participants who inconsistently or did not use condoms were less likely to be diagnosed with a syndromic STI is likely due to social desirability bias. A possible explanation for the finding that participants who considered themselves at risk of HIV were less likely to be syndromically diagnosed with an STI is that the realization of HIV risk might have influenced a positive change in their sexual behavior, which led to reduced STIs. In contrast, participants who reported having sex under the influence of alcohol or drugs and those who recurrently used PEP in this study were more likely to be diagnosed with a syndromic STI. These findings are plausible because alcohol consumption is associated with risky sexual behavior [27], and recurrent users of PEP are possibly involved in risky sexual behavior. The finding that AGYW who participated in safe space interventions had a higher likelihood of a syndromic STI diagnosis was expected because one of the eligibility criteria for participation in the DREAMS program is having been diagnosed or treated for an STI. This could be why the AGYW were enrolled in the safe space in the first place.

Based on the findings of this study, we recommend that the programs provide more education on safe sexual practices and the consistent use of condoms to ensure that AGYW do not contract STIs. Furthermore, we recommend that high‐risk AGYW be offered more counseling on STI prevention measures and safe sexual practices. A national study should be conducted to determine the prevalence of asymptomatic STIs among AGYW using the NAATs, as they also increase the risk of HIV acquisition. Furthermore, periodic sentinel surveillance among AGYW should be conducted to ensure the country has STI data for this population group. The findings of this study cannot be generalized to all AGYW, as those who participated in the DREAMS and Reach PHN programs were primarily high‐risk individuals. The responses used in this analysis were self‐reported and may have been subject to social desirability bias. The accuracy of adolescents’ self‐reports of their health risk behaviors depends on several factors, such as the ability to understand the questions, the ability to recall behaviors, the perceived social desirability of certain behaviors, motivation to complete surveys accurately, and the salience of the behavior to current/changing identity [28]. However, the findings provide valuable information to improve program services and reduce STIs among AGYW.

## 5. Conclusion

The study found that the prevalence of syndromic STIs among AGYW was 11.4%. Participants aged 15–19 years, with children, enrolled in 2022–2023, in programs for 13 months or longer, who were pregnant or breastfeeding, using a family planning method, or who perceived themselves at risk of HIV, exhibited a lower likelihood of having a syndromic STI diagnosis. Recurrent PEP users, individuals who have experienced sexual violence, and those who engaged in sexual activity while under the influence of alcohol or drugs exhibited a higher likelihood of syndromic STI diagnosis. It is recommended that the program needs to intensify education on safe sexual practices, promote the consistent use of condoms, offer individualized counseling to high‐risk AGYW to prevent STIs, and continue to strengthen the syndromic management of STIs. It is also high time to begin using laboratory and point‐of‐care systems for the molecular diagnosis of STIs among AGYW, as most infections are asymptomatic.

## Author Contributions

Enos Moyo: conceptualization; data analysis; and writing–original draft.

Endalkachew Melese: conceptualization and writing–review and editing.

Hadrian Mangwana: data abstraction and writing–review and editing.

Simon Takawira: data abstraction and writing–review and editing.

Bernadette Harases: writing–review and editing.

Rosalia Indongo: writing–review and editing.

Perseverance Moyo: writing–review and editing.

Ntombizodwa Makurira Nyoni: writing–review and editing.

Tafadzwa Dzinamarira: writing–original article and writing–review and editing.

## Funding

The PEPFAR, through USAID, funded the DREAMS project, the Reach PHN activity, and the present study.

## Disclosure

The abstract for this manuscript was presented at the 2025 INTEREST conference.

## Conflicts of Interest

The authors declare no conflicts of interest.

## Data Availability

The dataset associated with this manuscript can be provided on request from the lead author.
